# New Insights on the Regulation of the Insulin-Degrading Enzyme: Role of microRNAs and RBPs

**DOI:** 10.3390/cells11162538

**Published:** 2022-08-16

**Authors:** Yolanda Martín-Martín, Ana Pérez-García, Marta Torrecilla-Parra, Mario Fernández-de Frutos, Virginia Pardo-Marqués, María José Casarejos, Rebeca Busto, Cristina M. Ramírez

**Affiliations:** 1 IMDEA Research Institute of Food and Health Sciences, 28049 Madrid, Spain; 2 Servicio de Neurobiología, Instituto Ramón y Cajal de Investigación Sanitaria (IRYCIS), Hospital Universitario Ramón y Cajal, 28034 Madrid, Spain; 3 Servicio de Bioquímica-Investigación, Hospital Universitario Ramón y Cajal, IRYCIS, 28034 Madrid, Spain

**Keywords:** insulin, insulin-degrading enzyme (IDE), diabetes, Alzheimer’s disease (AD), RNA binding proteins (RBPs)

## Abstract

The evident implication of the insulin-degrading enzyme (IDE) in Alzheimer’s disease (AD) and type 2 diabetes mellitus (T2DM), among its capacity to degrade insulin and amyloid-β peptide (Aβ), suggests that IDE could be an essential link in the relation between hyperinsulinemia, insulin resistance and AD. However, little is known about the cellular and molecular regulation of IDE expression, and even less has been explored regarding the post-transcriptional regulation of IDE, although it represents a great molecular target of interest for therapeutic treatments. We recently described that miR-7, a novel candidate for linking AD and T2DM at the molecular level, regulates IDE and other key genes in both pathologies, including some key genes involved in the insulin signaling pathway. Here, we explored whether other miRNAs as well as other post-transcriptional regulators, such as RNA binding proteins (RBP), could potentially participate in the regulation of IDE expression in vitro. Our data showed that in addition to miR-7, miR-125, miR-490 and miR-199 regulate IDE expression at the post-transcriptional level. Moreover, we also found that IDE contains multiple potential binding sites for several RBPs, and a narrow-down prediction analysis led us to speculate on a novel regulation of IDE by RALY and HuD. Taken together, these results demonstrate the novel players controlling IDE expression that could represent potential therapeutical targets to treat several metabolic diseases with a high impact on human health, including AD and T2DM.

## 1. Introduction

Insulin resistance in the brain is a key pathological feature contributing to obesity, diabetes and neurodegenerative diseases. Insulin and insulin-like receptors (INSR, IGFRs) and signaling partners are distributed throughout the brain. In general, in the central nervous system (CNS), insulin and IGFs also play a crucial role in learning and memory, regulating processes such as neuronal stem cell activation, cell growth, synaptic maintenance and amyloid-β (Aβ) degradation. Interestingly, human preclinical studies have shown that Alzheimer’s disease (AD) is a degenerative metabolic disease, which is characterized by impairments in brain insulin responsiveness, glucose and energy homeostasis; it is therefore known as type 3 diabetes [1,2,3]. AD patients have shown brain insulin resistance, accompanied by reduced mRNA and protein expression of INSR, insulin receptor substrate (IRS) and IGF-1R [4,5]. Importantly, insulin can modulate the clearance of extracellular Aβ oligomers through regulating the insulin-degrading enzyme (IDE), which represents a key molecular link between AD and type 2 diabetes mellitus (T2DM) [6]. IDE is an atypical zinc-metalloprotease, recently shown to interact with proteasome components [7], widely known to prevent the formation of peptide aggregates by the cleavage and inactivation of several bioactive peptides, such as insulin, glucagon, amylin and Aβ [8,9,10,11]. Expectedly, an increase in insulin concentration may inhibit the degradation of Aβ through competition as a target for IDE, favoring Aβ deposits as a hallmark of AD [6,8,11,12]. IDE expression, which has been shown to influence Aβ levels in vitro and in vivo [13], is also impaired in the brains of AD patients and in Tg2576 transgenic mice and correlates with the accumulation of Aβ during the progression of the disease [14]. Moreover, IDE knockout (KO) mice display hyperinsulinemia, hyperglycemia, insulin and glucose intolerance, increased body mass and reduced levels of insulin receptors, as well as accumulation of endogenous Aβ [14,15,16].Importantly, human genetic studies have pinpointed the IDE region of Chr 10 with both T2DM and AD [16,17,18]. Therefore, IDE is a strong candidate link between both T2DM and a late onset of AD and represents an attractive therapeutical target. It has been previously suggested that controlling IDE levels could provide yet another potential therapeutic approach in aberrant metabolic states, and several studies have investigated the possibility of targeting IDE to prevent insulin degradation [8,19,20,21]. There is great interest in studying the mechanisms that govern the regulation of IDE expression and function as a target for therapeutic intervention [22]. Recent report has shown the novel regulators of IDE, such as JDS-chromium-insulin (CRI)-003, a novel form of insulin that has been directly conjugated with chromium instead of zinc [23]. Although IDE is regulated transcriptionally by insulin, to date, only a few studies [14] have explored the regulation of IDE at the post-transcriptional level.

MicroRNAs (miRNAs) are small (18–25 ntds), evolutionarily conserved, non-coding RNAs that have an important function in gene regulation, acting at the post-transcriptional level. By binding to the 3′UTR of target messenger RNAs (mRNAs), miRNAs repress translation or induce mRNA degradation, or both [24,25]. Interestingly, the expression of many of these miRNAs is altered in aberrant metabolic states, such as T2DM, as well as in AD [26,27,28]. miRNAs are critical regulators of glucose metabolism by regulating insulin metabolism in the pancreas and peripheral tissues, contributing to obesity and diabetes [29]. Previous studies from our laboratory have reported that miR-7, a miRNA enriched in neuroendocrine tissues, such the pancreas and brain, targets and represses several components of the insulin pathway, such as INSR and IRS2, together with important regulators of AD, including IDE [30]. Despite the role of miRNAs in regulating insulin resistance in the brain and in AD remains unexplored. Thus, considering the relevance of IDE in this context, we aimed to explore additional post-transcriptional regulators that could contribute to influencing IDE expression.

In addition to non-coding RNAs, RNA binding proteins (RBPs) are a heterogeneous group of proteins that play a major role in gene expression by controlling all stages of RNA biology, from transcription, RNA processing, alternative splicing, mRNA stability and mRNA localization to translation and RNA degradation, via specific RNA interaction motifs generally present in the 3´UTR of the target mRNA [31,32]. As we reviewed, several RBPs have been shown to regulate metabolism and take part in insulin resistance [33]. However, until now, there has been no evidence that RBPs can potentially bind to IDE transcript or even regulate its levels of expression.

Taking all this into account, here, we identified miR-125-5p, miR-31-a, miR-199-3p and miR-490-3p as potential candidates to target IDE 3′UTR, in addition to miR-7, as we previously reported [30]. Using bioinformatic prediction algorithms and gene ontology (GO) enrichment analysis, we found that miR-31 and miR-199 may regulate IDE and could be two potential targets in developing novel therapies for AD and T2DM. In addition, we studied the possible influence of different RBPs on the IDE 3′UTR. Importantly, we showed that RALY and ELAVL4 (HuD) present a number of potential binding sites to the human IDE 3′UTR.

## 2. Materials and Methods

### 2.1. Bioinformatic Analysis of miRNAs and RBPs Target Genes

The predicted target miRNAs for IDE 3′UTR were identified using TargetScan 8.0. (http://www.targetscan.org, accessed on 26 May 2022) [34]. To confirm the prediction, we used four other different miRNA computational algorithms (miRDB, miRWalk, mirDIP and miRmap) that utilize distinct parameters to predict the probability of functional miRNA binding site [24]; we analyzed the results obtained using the Venn diagram. The predicted human target genes for miR-31-5p and miR-199a-3p were identified using TargetScan 8.0. [34], and the genes were analyzed by gene ontology using Panther v8.0 (http://www.pantherdb.org, accessed on 22 June 2022) [35]. For a further study of miR-31, the predicted targets were uploaded and analyzed by DAVID (https://david.ncifcrf.gov, accessed on 22 June 2022) [36,37].

Moreover, we predicted the target genes for hIDE using RBPmap (http://rbpmap.technion.ac.il/, accessed on 26 May 2022) [38]. Putative targets with more than 100 target sites were uploaded into the Panther v8.0 gene classification system (http://www.pantherdb.org, accessed on 22 June 2022) [35] to analyze pathway enrichment.

### 2.2. Cell Culture and Treatments

The human neuroblastoma SH-SY5Y (SH) cells and human hepatic cell line (HepG2), obtained from the American Type Culture Collection, were maintained in Dulbecco’s modified eagle medium (DMEM) supplemented with 10% fetal bovine serum (FBS), 2% penicillin-streptomycin and L-glutamine in 10 cm^2^ dishes at 37 °C with 5% CO_2_. SH cells are a fast-growing neuroblastoma cell line from human that has been widely used to study neurotoxicity, metabolism, miRNAs and AD.

### 2.3. Transfection of miRNA Mimics, miRNA Inhibitors and siRBPs

Cells (~70% confluence) were transfected with 40 nM miRIDIAN miRNA mimic (miR-7-5p, miR-31-5p, miR-490-3p and miR-199a-3p) (Dharmacon; Lafayette, CO, USA); mirVana™ miRNA Mimic (miR-125-5p) (Ambion; Austin, TX, USA; Waltham, MA, USA); or with 90 nM miRNA hairpin miRNA inhibitor (Inh-miR-31-5p) (Dharmacon) by utilizing Lipofectamine RNAiMax or Lipofectamine 2000 (Invitogen; Waltham, MA, USA) and studied 24 h later for miRNA mimics or 48 h later for miRNA inhibitor. For RBPs experiments, 60 nM of siHuD and siRALY (Dharmacon) was transfected using Lipofectamine 2000. In all experiments, an equal concentration of a non-targeting control mimic (CM), control inhibitor (CI) or non-silencing control (NS), respectively, was used as a control for non-sequence-specific effects in miRNA and RBPs experiments. Verification of miRNAs overexpression was performed using quantitative PCR (qPCR), as described below.

### 2.4. 3‘UTR Luciferase Reporter Assays

cDNA fragments corresponding to the 3′UTRs of the human IDE gene were amplified by reverse-transcription PCR from genomic DNA with XhoI and NotI linkers. The PCR products were directionally cloned downstream of the Renilla luciferase open reading frame in the psiCHECK2 vector (Promega; Madison, WI, USA), which also contains a constitutively expressed firefly luciferase gene, which is used to normalize transfections. Site-directed mutations in the seed region of predicted miR-7-5p, miR-125-5p, miR-31-5p, miR-490-3p and miR-199a-3p sites within the 3′UTRs were generated using a QuikChange multisite-directed mutagenesis kit (Agilent; Santa Clara, CA, USA) according to the manufacturer’s protocol. All constructs were confirmed by sequencing. HEK-293 cells were plated into 12-well plates and co-transfected with 1 μg of the indicated 3′UTR luciferase reporter vectors and the mimics or negative-control mimic (Dharmacon) for the miRNA assay and siRNAs or non-silencing control (NS) for the RBPs assay using Lipofectamine 2000 (Invitrogen). Luciferase activity was measured using the Dual-Glo luciferase assay system (Promega). Renilla luciferase activity was normalized to the corresponding firefly luciferase activity and plotted as a percentage of the control (CM). The experiments were performed in triplicate.

### 2.5. RNA Isolation and Quantitative Real-Time PCR

Total RNA was isolated from cells using the Trizol reagent (Invitrogen) according to the manufacturer’s protocol. For mRNA quantification, 1 μg of total RNA was reverse transcribed to cDNA using the iScript RT supermix (Bio-Rad; Hercules, CA, USA), according to the manufacturer’s protocol. Quantitative real-time PCR was performed in triplicate using the iQ SYBR green supermix (Bio-Rad) on a real-time detection system (Eppendorf; Hamburg, Germany). The mRNA level was normalized to the level of GAPDH rRNA as a housekeeping gene. The primer sequences are available upon request. For miRNA quantification, total RNA was reverse transcribed using the miScript II RT kit (Qiagen; Hilden, Germany).

### 2.6. Western Blot Analysis

Cells were lysed in ice-cold buffer containing 50 mM Tris-HCl (pH 7.4), 0.1 mM EDTA, 0.1 mM EGTA, 1% NP-40, 0.1% sodium deoxycholate, 0.1% SDS, 100 mM NaCl, 10 mM NaF, 1 mM sodium pyrophosphate, 1 mM sodium orthovanadate, 1 mM Pefabloc and 2 mg/mL protease inhibitor cocktail (Roche Diagnostics Corp.; Basel, Switzerland). Protein concentrations were determined using the DC protein assay kit (Bio-Rad; Hercules, CA, USA). Cell lysates containing 12.5 to 25 μg of protein were analyzed by SDS-PAGE and immunoblotting. The primary antibodies used included antibodies to IDE (catalog number ab32216; Cell Signaling), RALY (catalog number ab170105; Abcam; Cambridge, United Kingdom), HuD (catalog number ab171448; Abcam) and heat shock protein 90 (HSP90) (catalog number 610419; BD Biosciences; Franklin Lakes, NJ, USA). Secondary antibodies were fluorescence-labeled antibodies, and bands were visualized using the Odyssey infrared imaging system (Lincoln, NE, USA). Densitometry analysis of the Western blots was carried out by using the ImageJ software from the NIH (http://rsbweb.nih.gov/ij/, accessed on 8 July 2022).

### 2.7. Mouse Studies

Male 5xFAD mice (MMRRC Strain #034848-JAX; Strain name: B6.Cg-Tg(APPSwFlLon, PSEN1*M146L*L286V) 6799Vas/Mmjax) at 9 months of age (n = 3) were purchased from Jackson Laboratories (Bar Harbor, ME) and kept under constant temperature and humidity in a 12 h controlled dark/light cycle in the animal facility of The Ramon y Cajal Universitary hospital (IRYCIS). Age-matched wild-type mice were used as controls. Mice were given ad libitum access to water and food. All animal procedures were carried out in accordance with the National Institutes of Health guide for the care and use of laboratory animals guidelines approved by The Ethics Committee of Animal Research of the Hospital Ramón y Cajal and Madrid Government. Mice were sacrificed by CO_2_, and brain tissue samples were dissected and stored at −80 °C until processing for histochemistry and protein expression analysis.

### 2.8. Thioflavin-S Staining

Mice brain tissues were sacrificed by cervical dislocation, and the extracted brains were fixed in 4% paraformaldehyde solution (Quimigen; Madrid, Spain). After 24 h of fixation, brains were immersed in 30% sucrose for 2 days For Thioflavin-S staining, 10 µm thick frozen sections were incubated for 2 h at 60 °C. Then, the slides were rehydrated in xylene and ethanol solutions (100%, 90% and 70%). After washing in water, the slides were submerged in Thioflavin S for 3 min and then rinsed in water and 1% acetic acid. Stained slides were mounted on aqueous fluoromount, and images were captured with a Nikon A1R confocal microscope.

### 2.9. Statistical Analysis

In vitro experiments were routinely repeated at least three times. Data are expressed as standard errors of the means (SEM) unless otherwise indicated. Statistical differences were measured using unpaired two-sided Student’s *t*-test. Normality was checked using the Kolmogorov–Smirnov test. A *p* value of ≤0.05 was considered statistically significant. Data analysis was performed using GraphPad Prism 8.0.1 (GraphPad, San Diego, CA, USA).

## 3. Results

### 3.1. Several miRNAs Potentially Bind to hIDE 3′UTR

**Figure 1:**
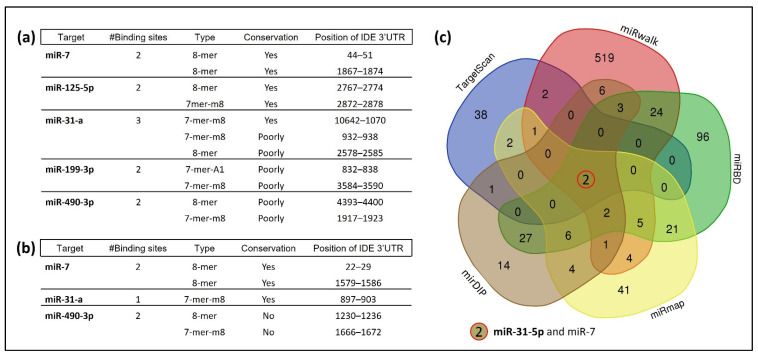
Prediction analysis of IDE target by multiple miRNAs. (**a**) Predicted human target miRNAs of IDE, showing the number and type of binding sites and their conservation. (**b**) Predicted mouse target miRNAs of IDE, showing the number and type of binding sites and their conservation. (**c**) Venn diagram depicting hIDE targeting miRNAs predicted by five different bioinformatic databases.

miRNAs can regulate gene expression by binding to target mRNAs and suppressing their translation or initiating their degradation. To define possible miRNA–IDE mRNA interactions, we first investigated which miRNA families could target hIDE 3′UTR and regulate its expression. To achieve this, we carried out an exhaustive bioinformatic analysis using TargetScan 8.0. to predict the IDE target miRNAs in mice and humans. We explored miRNA families broadly conserved among the vertebrates, finding 47 families that presented at least one complementary site within human IDE mRNA. From these, five miRNAs, miR-7, miR-31-a, miR-125-5p, miR-199a-3p and miR-490-3p, were selected based on their involvement in insulin resistance [26,30,39,40]. Figure 1a,b show the number, type and conservation of predicted sites for the selected miRNAs. Among them, miR-7, miR-31-a and miR-490-3p are conserved between mice and humans. Later, four additional computational algorithms (miRDB, miRWalk, mirDIP and miRmap) confirmed the initial prediction analysis. Further analysis using a Venn diagram showed miR-7 and mir-31-5p as the top common predicted miRNAs that could target hIDE 3′UTR (Figure 1c).

### 3.2. Post-Transcriptional Regulation of hIDE by miRNAs

**Figure 2:**
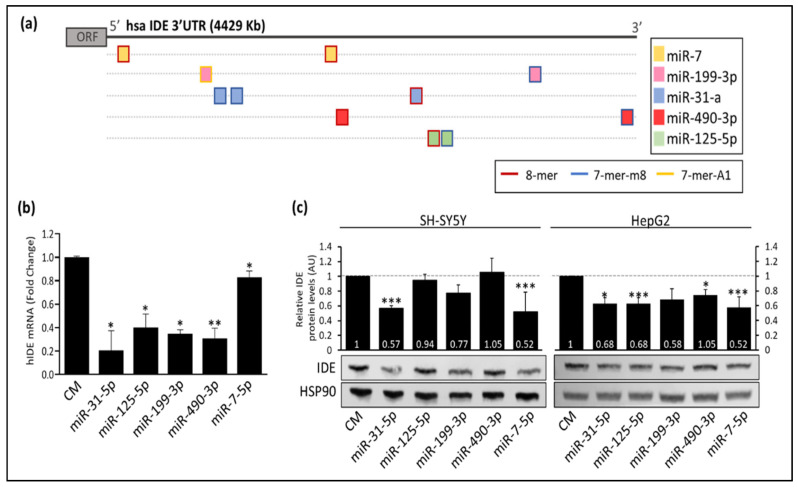
The expression of IDE is regulated by multiple miRNAs in vitro. (**a**) Representation of predicted target miRNAs sites for hIDE 3′UTR. The square flange color indicates the type of binding site. The color inside the square shows the miRNA targets of that region (**b**) qPCR of IDE in SH cells. (**c**) Representative Western blot analysis of IDE in SH and HepG2 cells transfected with miR-31-5p, miR-125-5p, miR-199a-3p and miR-7-5p. (Top) Relative hIDE levels corresponding to the means ± SEM from three independent experiments. *, *p* < 0.05; **, *p* < 0.01; ***, *p* < 0.001 (significantly different from cells transfected with the CM).

Based on these findings, we decided to further explore the potential targets of hIDE 3′UTR. We found that the miRNAs binding sites were distributed along the hIDE 3′UTR (Figure 2a). The potential targets sites and types for miR-7, miR-199-3p, miR-31-a, miR-490-3p and miR-125-5p are represented in Figure 2a. Then, we assessed the IDE mRNA expression and protein levels upon overexpression of these miRNAs in SH cells. As shown in Figure 2b, the IDE mRNA was significantly downregulated by the overexpression of these miRNAs. Similar results were found for protein levels (Figure 2c). While changes in miR-125-5p, miR-199a-3p or miR-490-3p overexpression were subtle, miR-31-5p and miR-7-5p overexpression presented the most potent downregulation of IDE levels in SH cells by Western blot (Figure 2c).

**Figure 3:**
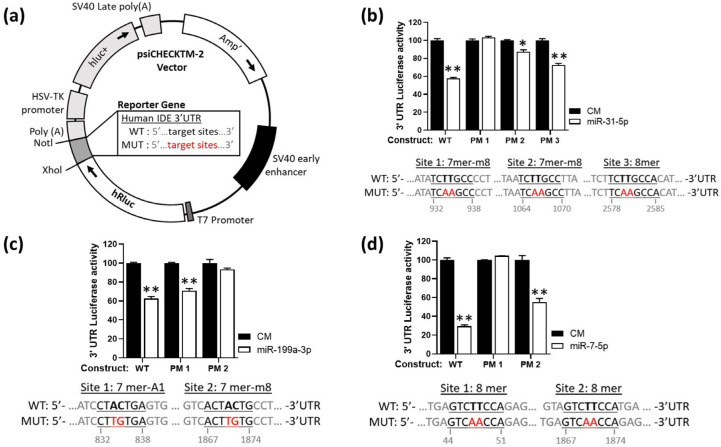
Human IDE 3′UTR sequence contains target sites for miR-31-5p, miR-199-3p and miR-7-5p. (**a**) psiCHECKTM-2 vector representation. (**b**–**d**) Underlined sequences indicate the miRNAs binding sites. Nucleotides highlighted in red indicate the point mutations in the miRNAs binding sites. Luciferase reporter activity in HEK-293 cells transfected with the CM or miRNAs mimic and the IDE 3′UTRs (wild-type (WT)) or the constructs containing the indicated point mutations (PM). Data are expressed as relative luciferase activities compared to the activity in control samples co-transfected with an equal concentration of the CM and correspond to the mean SEM from three experiments performed in triplicate. * *p* < 0.05, ** *p* < 0.01 (significantly different from cells co-transfected with CM and the WT or PM 3′UTR).

Given these results, we next sought to investigate the potential direct binding of these miRNAs to hIDE 3´UTR. To do so, we cloned the full hIDE 3′UTR (4Kb) into a dual luciferase reporter plasmid (Figure 3a) and assessed their activity under miR-7-5p, miR-31-5p or miR-199a-3p overexpression conditions (Figure 3b–d). These experiments demonstrated a significant reduction in the hIDE 3′UTR activity under overexpression conditions for miR-7-5p, miR-31-5p or miR-199a-3p. The point mutations potentially relieved the inhibition effect of miR-7-5p, miR-31-5p or miR-199a-3p, indicating that modulation at the post-transcriptional level is due to the binding of these miRNAs to the hIDE 3′UTR, at least partially (Figure 3b–d). Similar experiments were performed for miR-125-5p and miR-490-3p (Figure S1).

### 3.3. miR-31 and miR-199 Target Key Genes Involved in AD

**Figure 4:**
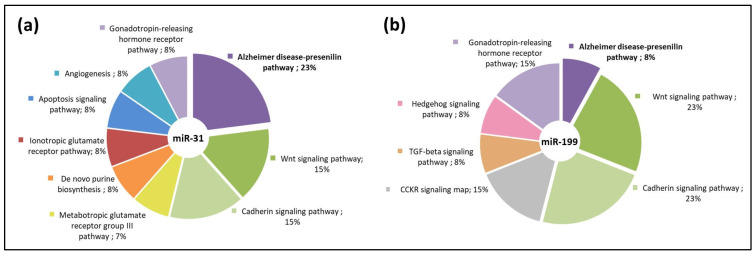
Bioinformatic analysis of predicted miR-31 and miR-199 target genes. Gene ontology analysis using the Panther software showing enriched pathways for miR-31 predicted target genes (**a**) and miR-199 predicted target genes (**b**).

To explore whether the molecular pathways would be affected by miR-31 and miR-199, we performed a predicted target gene analysis using bioinformatic tools for miRNA target predictions [34]. We analyzed the predicted target genes (Figure 4) that contain at least one 8-mer binding site using gene ontology [35].

**Figure 5:**
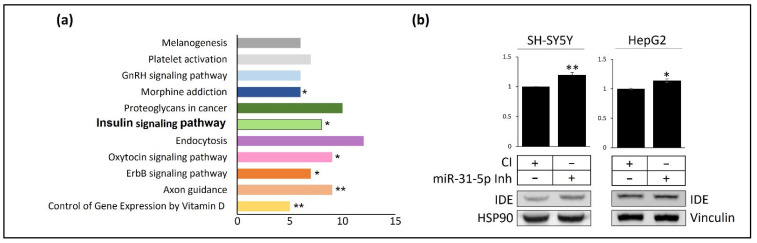
miR-31-5p regulates genes involved in insulin signaling pathway and controls IDE protein at the physiological level (**a**) DAVID analysis showing fractional difference analysis of the top enrichment pathways of miR-31-5p target genes. * *p* < 0.05; ** *p* < 0.01 (observed versus expected). (**b**) Representative Western blot analysis of IDE in SH and HepG2 cells transfected with control inhibitor (CI) or miR-31 inhibitor. HSP90 and Vinculin were used as loading controls.

In addition, to further study the miR-31 function, we analyzed miR-31 target genes by a gene enrichment analysis using DAVID. From the analysis, miR-31 showed the highest enrichment in AD-related genes compared to the rest of the overrepresented pathways (Figure 5a). Moreover, we decided to explore whether miR-31-5p could have a direct implication for IDE expression at the physiological level. For this purpose, we used specific inhibitors of miR-31-5p in two cell lines (SH and HepG2). We found that inhibiting the expression of mir-31-5p increased IDE protein levels, corroborating that IDE is regulated by miR-31-5p (Figure 5b).

**Figure 6:**
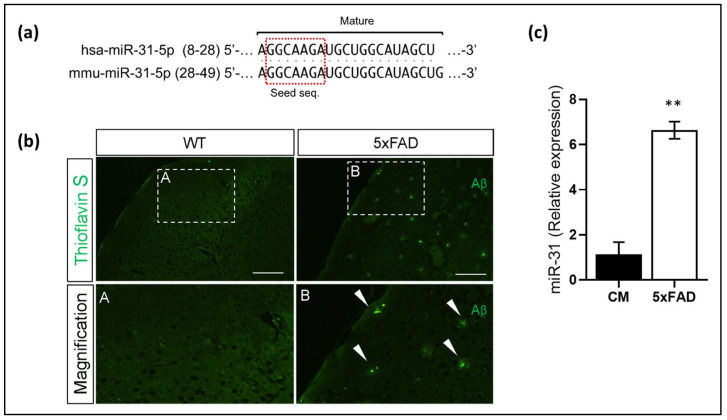
miR-31 could play an interesting role in AD. (**a**) Representation of the mature sequences of miR-31-5p in humans and mice. (**b**) Representative images of cortical Aβ aggregates in mice stained with Thioflavin S. Scalebar: 10 μm (**c**) qPCR of miR-31-5p in mouse cortex. Relative miR-31 expression corresponding to the means ± SEM from three independent experiments. ** *p* < 0.01; (significantly different from cells transfected with the CM). A and B represent magnifications images of insets selected in WT and 5xFAD, respectively.

The MiR-31-5p mature sequence is highly conserved between the mouse and the human (Figure 6a). Finally, we decided to explore the expression of miR-31 in AD. To do so, we used a humanized 5xFAD transgenic mice model, which express human APP and PSEN1 transgenes with a total of five AD-linked mutations. These mice showed a marked accumulation of Aβ aggregates compared to WT (Figure 6b). Interestingly, our qPCR analysis of miR-31 in mouse cortex showed increased levels of this miRNA in the cortex of 5xFAD compared to WT mice (Figure 6c).

### 3.4. Post-Transcriptional Regulation of hIDE 3′UTR by RBPs

RBPs can control the fate of transcripts through mRNA binding. Since no data showed the regulation of IDE by RBPs and the role of these proteins in insulin signaling, we decided to explore this novel possibility. By using the prediction algorthim RBPmap [38], we found 130 RBPs with at least one binding site in the hIDE 3′UTR. From those, we narrowed down the RBPs list by selecting those containing more than 300 consensus sites, theoretically selecting those with a higher probability of binding to hIDE 3′UTR (Table 1).

**Figure 7:**
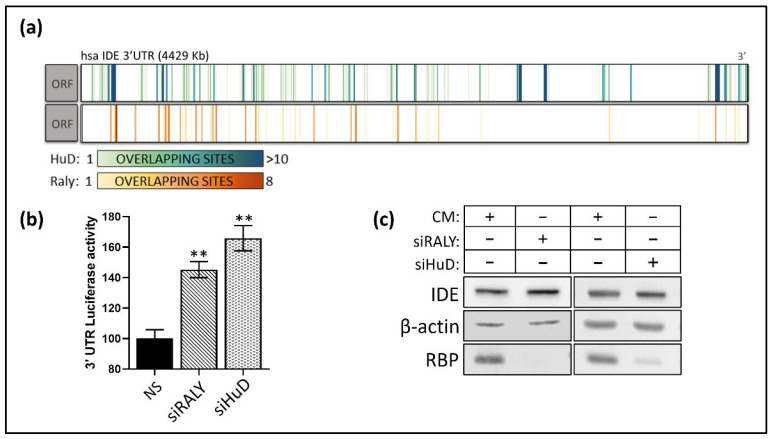
RALY and HuD are post-transcriptional regulators of IDE. (**a**) Representation of predicted target RALY and HuD sites for human IDE 3′UTR. (**b**) Luciferase reporter activity in HEK-293 cells transfected with the NS or siRNA against HuD or RALY and the IDE 3′UTRs. Data are expressed as relative luciferase activities compared to the activity in control samples co-transfected with an equal concentration of NS. ** *p* < 0.01 (significantly different from cells co-transfected with NS and the siRBPs). (**c**) Western blot analysis of IDE in SH cells transfected with the respective RBP silencing.

Among the RBPs examined, we focused on two of them, RALY and HuD, due to their involvement in pathways related to metabolism and neurodegeneration [63,64,65,66]. We further performed an analysis of the location and density of the overlapping binding sites and the selected RBPs at the 3′UTR of IDE, revealing several parts of this region with a high number of overlapping consensus sequences through which these RBPs could interact with IDE mRNA (Figure 7a). To further explore the potential effect of these RBPs on IDE, we performed 3′UTR luciferase assays of human IDE in previously transfected cells treated with specific siRNAs against RALY or HuD. This analysis showed that the silencing of RALY and HuD significantly increases IDE 3′UTR luciferase activity (Figure 7b), which correlated with an increase in IDE protein levels under the same conditions (Figure 7c). Although further studies are needed to confirm the outcome of the effects of the binding of these RBPs to IDE, these initial observations point out the potential new targets, including two new miRNAs, for IDE regulation at the post-transcriptional level.

## 4. Discussion

Brain insulin resistance and insulin deficiency are mediators of cognitive impairment and neurodegeneration, which support a pathophysiological connection between AD and diabetes [2,5,11,67,68]. Despite this, the molecular mechanism underlying this relation remains unclear. IDE has been proposed as an essential link between both pathologies due to its role in insulin and extracellular Aβ oligomers clearance [67,69]. For this reason, IDE is a promising therapeutical target, and understanding the underlying mechanisms of its regulation may be the key to the development of new therapies. Little is known about the physiological regulation of IDE expression and its activity in the brain. Instead, IDE regulation has been studied in hepatocytes because of its role in insulin modulation. There is evidence that IDE activity increases through insulin exposure, while its mRNA and protein levels remain unaltered in hepatocytes [15,70]. Interestingly, exogenous insulin upregulates IDE protein levels in mouse primary hippocampal neurons [70]. In addition to insulin regulation, there are several studies that demonstrate that IDE is regulated at the transcriptional level [28].

It has been described that chelators, divalent cations, insulin-binding inhibitors could block IDE function, and other biomolecules, such as non-esterified fatty acids, nucleotide triphosphates [35], post-translational modification and protein interaction, can also modulate its activity [36]. Indeed, the discovery of the crystal structure of IDE opens the door to novel pharmacological regulators of IDE functions [70]. Although miRNAs are abundantly expressed in the brain, where they are involved in the modulation of multiple physiological functions and pathological states and metabolic pathways involved in neurodegeneration [24,25,33,71], only a few studies have explored the potential post-transcriptional regulation of IDE [28].

The first miRNA that has been described as a modulator of IDE expression was miR-7, which is an important post-transcriptional regulator of brain insulin resistance and AD. We described that miR-7, which is itself regulated by insulin, impairs insulin signaling and could lead to insulin resistance in the brain through the post-transcriptional regulation INSR, IRS-2. In addition to insulin signaling, miR-7 can also target genes directly involved in AD, such as IDE, together with other neuroprotective pathways against AD, such as the LXR-ABCA1 axis involved in Aβ metabolism [30]. In this work, we explored additional miRNAs, such as miR-199a-3p and miR-31-a, in the regulation of IDE expression.

miR-31 is highly expressed in neural stem cells (NSCs), where it plays an essential role in NCSs division and proliferation [72], and it was found to be required for terminal astrocyte differentiation, development and maturation through the control of Lin28 levels [73]. Moreover, miR-31 improves the functional recovery after ischemia stroke through the inhibition of neuron apoptosis [74]. Therefore, it is not surprising that miR-31 dysregulation can contribute to neurological dysfunctions. Indeed, several groups have described altered levels of miR-31 in plasma from AD patients [40,75]. However, there are no data available showing the changes of miR-31 levels in susceptible target tissues in AD, such as the cortex or hippocampus, compared to healthy tissues. It would be of interest to determine whether there is an inverse correlation between the expression of IDE and miR-31, as was found in human brain AD for miR-7 [30]. On the other hand, miR-199 has also been implicated in crucial functions in the brain, such neurogenesis and neuronal migration [71,76,77]. In particular, miR-199 levels increase during early brain development and modulate extracellular signal-regulated kinase (ERK) [76]. Intriguingly, an in silico analysis reported that AD patients’ brains present upregulated levels of miR-199 [78]. Song et al., showed that miR-199 was involved in the development of AD by decreasing the expression of neuritin, a protein involved in neural development and plasticity [77]. Moreover, miR-199a-3p can also impair the autophagic process, leading to the accumulation of autophagosomes and accumulation Aβ [79]. These findings highlight the importance of miR-31 and miR-199 in the correct development and function of the brain. However, to our knowledge, this is the first report describing the modulation of IDE by these two miRNAs. In the present study, we demonstrated, for the first time, that miR-199a-3p and miR-31-a significantly decreased IDE levels (Figure 2). By using luciferase reporter vectors containing the WT hIDE 3′UTR and mutated constructs, we verified that miR-31-a and miR-199a exert inhibitory effects on IDE expression due to their direct binding to IDE mRNA, at least partially (Figure 3). We also examined whether miR-31 target genes were implicated in any pathway related to IDE biological functions, finding an enrichment in AD-presenile pathways. Although the case of miR-199 showed less involvement in this pathway, Aranda et al., demonstrated that miR-199 targets genes involved in intracellular trafficking, in addition to Caveolin-1 [71,80], which could impair insulin signaling through dysregulation of INSR, which is primarily located in the lipid raft. Consequently, it can be hypothesized that miR-199 could indirectly modulate IDE transcriptional regulation by insulin. On the other hand, our analysis showed that miR-31 could target other genes involved in the insulin signaling pathway, such as IRS. Again, this could highlight an alternative way for the regulation of IDE levels by miR-31. Considering the negative feedback mechanism by which insulin activates the insulin receptor and upregulates IDE in a phosphoinositide 3-kinase (PI3K)-dependent way [14], miR-31 could alter this pathway by targeting IRS, an PI3K upstream molecule, and consequently modify IDE levels. More experiments are needed to better characterize the potential underlying mechanisms that could allow miR-31 and miR-199 to post-transcriptionally regulate IDE.

In addition to miRNAs, we aimed to explore the possible post-transcriptional regulation of IDE by RBPs. As shown, IDE has a relatively long 3′UTR, which makes it highly susceptible to modification at the post-transcriptional level. This is also the case for other targets involved in metabolism and AD, such as ABCA1, whose post-transcriptional regulation by miRNAs and RBPs has been extensively explored [80,81,82,83,84]. Firstly, we assessed a prediction analysis of all the RBPs that contained at least one consensus site along the hIDE 3′UTR. From them, we selected those RBPs that showed more than 300 consensus sites (Table 1). RALY and HuD attracted our attention because of their involvement in metabolism and neurodegeneration [63,64,65,66]. However, in vitro analyses are needed to confirm this prediction of direct binding of these RBPs to the hIDE 3′UTR or, alternatively, by a competing mechanism with other post-transcriptional regulators. Indeed, several examples in the literature have demonstrated the interplay between miRNAs and RBPs [33]. Our data suggest a potential novel regulatory mechanism through miRNAs and RBPs that could be exploited as a therapeutical target in vivo. Future studies will help decipher the possible mechanisms underlying IDE regulation by these RBPs, which is even more important considering that little is known regarding RBPs functions in the context of insulin resistance in AD [33].

To summarize, a better understanding of IDE regulation would lead to therapeutical options for treating metabolic pathologies and AD. Here, we show, for the first time, that, in addition to miR-7, several other miRNAs can alter IDE expression. On the other hand, mild, post-transcriptional regulation of IDE expression cannot be dismissed, since it represents one of the most pursued therapeutical targets against AD and represents a key common element linking insulin resistance and AD.

## Figures and Tables

**Table 1:** Predicted target RBPs of hIDE 3’ UTR, showing the number and type of binding sites and their conservation in human.

Gene	Protein Name	N° Binding Sites	Matching Motif	RNA Motif	Biological Function	Reference
RALY	RNA-binding protein RALY	301	uuuuu/uuuuuub	RRM × 1	Cholesterol biosynthesis Mitochondrial metabolism	[41,42,43]
ELAVL4 (HuD)	ELAV-like protein 4	322	uuauu/uaauu	RRM × 3	mRNA metabolism regulation Post-transcriptional control of gene expression Nervous system development and plasticity	[44,45,46,47]
TIA1	Nucleolysin TIA-1 isoform p40	322	uuuuubk/uuuuu	RRM × 3	Synaptic plasticity regulation Alternative pre-RNA splicing Regulation of mRNA translation Apoptosis	[48,49]
RBMS3	RNA-binding motif, single-stranded-interacting protein 3	347	auauau/hauaua	RRM × 2	Tumor cell defense Gene expression regulation	[50,51,52]
DAZ3	Deleted in azoospermia protein 3	348	aguuu/ uuguuu	RRM × 1	Spermatogenesis	[53,54,55]
HNRNPC	Heterogeneous nuclear ribonucleoproteins C1/C2	349	huuuuuk/ uuuuu	RRM × 1	Cell cycle RNA Polymerase II Transcripts for Export	[56,57]
KHSRP/ FUBP2	Far upstream element-binding protein 2	355	uguau/ uuuuu	KH × 4	Transcriptional regulator Gene expression regulation	[58,59]
HNRNP CL1	Heterogeneous nuclear ribonucleoprotein C-like 1	357	huuuuuk/ uuuuu	RRM × 1	Nucleosome assembly	[60,61]
FUBP3	Far upstream element-binding protein 3	375	uauau/ uuaau/ uuuau	KH × 4	Gene expression	[62]

## Data Availability

The data presented in this study are available in the article.

## Supplementary Materials

The following supporting information can be downloaded at: https://www.mdpi.com/article/10.3390/cells11162538/s1, Figure S1: Human IDE 3′ UTR sequence contains target sites for miR-125-5p and miR-490-5p.
