# Supplementary material for: New Insights on the Regulation of the Insulin-Degrading Enzyme: Role of microRNAs and RBPs
Source: Cells. 2022 Aug 16;11(16):2538. doi: 10.3390/cells11162538 (PMC9406717; doi:10.3390/cells11162538)

cells MDPI

Supplementary Materials:

Supplementary Figure S1. Human IDE 3′ UTR sequence contains target sites for the miR-125-5p and miR-490-5p. (a) Underlined sequences indicate the miRNAs binding sites. Nucleotides highlighted in red indicate the point mutations in the miRNAs binding sites. (b) Luciferase reporter activity in HEK-293 cells transfected with the CM or miRNAs mimic and the hIDE 3′ UTRs (wild type [WT]) or the constructs containing the indicated point mutations (PM). Data are expressed as relative luciferase activities compared to the activity in control samples cotransfected with an equal concentration of the CM and correspond to the means SEM from three experiments performed in triplicate. *, P<0.05; **, P<0.01 (significantly different from cells cotransfected with CM and the WT or PM 3′UTR).

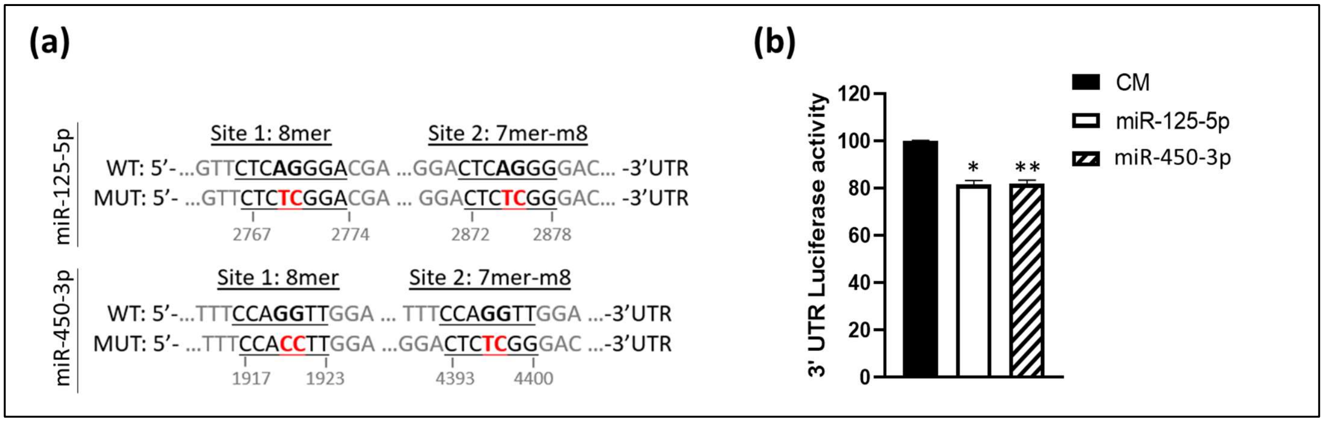

Supplement: Supplementary file 1 [file cells-11-02538-s001.zip › cells-1806668-supplementary.pdf]
